# Artificial Intelligence Use and Cognitive Resource Allocation: Nonlinear Associations with Mental Workload and Perceived Performance

**DOI:** 10.3390/jintelligence14080154

**Published:** 2026-07-31

**Authors:** Şahin Danişman, Filiz Evran Acar

**Affiliations:** 1 Department of Mathematics Education, Faculty of Education, Duzce University, Duzce 81620, Türkiye; 2 Department of Educational Sciences, Faculty of Education, Duzce University, Duzce 81620, Türkiye; filizacar@duzce.edu.tr

**Keywords:** artificial intelligence in education, pre-service teachers, mental workload theory, NASA-TLX, perceived workload, AI tool use, AI-supported pedagogical design

## Abstract

This study investigated how pre-service teachers’ frequency of artificial intelligence (AI) tool use is associated with their perceived workload and perceived task performance in AI-supported pedagogical design tasks, grounded in mental workload theory. Using a quantitative cross-sectional survey design, data were collected online from 464 pre-service teachers through a structured questionnaire covering AI use profiles, purposes of AI use, perceived usefulness of AI functionalities, and NASA-TLX-based workload ratings. Perceived workload was assessed using the raw NASA-TLX after participants completed an AI-supported lesson planning task involving the design of an instructional activity and teaching materials. Descriptive findings showed that AI use was widespread, with most participants reporting weekly or daily engagement. One-way ANOVA results indicated significant differences across AI use-frequency groups on all NASA-TLX dimensions. Infrequent users reported higher mental demand, temporal demand, effort, and frustration, as well as lower perceived task performance. However, polynomial trend analyses showed that the association was not strictly linear across all dimensions. Some workload dimensions were lowest among occasional users, whereas frustration decreased and perceived task performance increased as AI use frequency increased. These findings suggest that the workload implications of AI engagement may vary depending on the specific dimension of task demand considered.

## 1. Introduction

In today’s rapidly digitalizing and globalizing world, artificial intelligence (AI) technologies have emerged as a transformative force that is profoundly shaping the future of education. The integration of AI into learning processes holds the potential to redefine traditional pedagogical paradigms by offering personalized learning pathways and making learning more adaptive, inclusive, and effective. A large-scale global study conducted across 47 countries (n = 48,340) reported that 83% of students regularly use AI tools in their education (Gillespie et al., 2025). At the national level, research indicates that AI usage among undergraduate students in the United Kingdom reached 92% in 2025 (Freeman, 2025), while in the United States the proportion of higher-education students using AI on a daily or weekly basis increased from 14% in 2023 to 42% in 2025 (Shaw et al., 2025). Similarly, 20.95% of Chinese engineering students report daily AI use, and 41.89% report using AI several times a week (Fan et al., 2025), hence these figures suggest that AI is not merely a technological innovation but a structural force reshaping the nature of learning and academic work. Therefore, exploring how frequently artificial intelligence tools are used in educational contexts and examining their cognitive implications has become an essential focus for research on AI integration in learning environments (Asio & Gadia, 2024).

AI-supported tools provide personalized learning opportunities, enabling students to access information and learn at their own pace in alignment with their individual needs (Ayeni et al., 2024; Holmes et al., 2019; Mnguni, 2023). They have also been associated with improvements in academic achievement, learning efficiency, initiative, creativity, and independent thinking skills (Fan et al., 2025; Holmes et al., 2019). In addition, AI applications can assist teachers by reducing time spent on routine tasks, thereby allowing them to reorganize and refine their instructional practices (Shaw et al., 2025). However, the rapid expansion of AI in educational contexts has also raised questions about how educators perceive its adoption, including concerns about trust, transparency, and the conditions under which AI tools should be implemented in teaching and learning practices (Bilgin & Güngören, 2025). Questions have been raised regarding the accuracy and reliability of AI-generated outputs. The limited artificial intelligence literacy among higher-education students may hinder their capacity to critically evaluate AI-generated content (Sari et al., 2025), hence the risk of misleading or incorrect information may lead students to develop misconceptions, and the need to verify and evaluate AI-generated content can require additional cognitive effort (Fan et al., 2025). Moreover, excessive reliance on AI tools may contribute to shifts in learners’ cognitive engagement, potentially weakening independent thinking, critical analysis, and problem-solving processes (Fan et al., 2025; Gillespie et al., 2025; Pitts et al., 2025). Although students may perceive that they complete tasks more quickly with AI support, this perceived efficiency does not necessarily translate into genuine academic improvement or deep learning; rather, it may create an illusion of productivity that masks superficial understanding (Fan et al., 2025; Tsiani et al., 2025). These issues indicate that while AI can make some tasks easier, it can also bring new cognitive and metacognitive challenges. This raises important questions regarding how learners experience, allocate, and regulate their cognitive resources during AI-supported academic activities. Taken together, these contrasting findings suggest that the cognitive consequences of AI use are unlikely to be fully explained by a simple assumption of workload reduction. Rather, AI may simultaneously alleviate certain task demands while introducing new cognitive and metacognitive challenges that warrant further investigation.

This situation suggests that evaluating AI-supported learning processes solely on the basis of output speed or subjective performance perceptions may be insufficient. Rather than focusing on how quickly a task is completed, a more informative criterion lies in how the balance between task demands and the learner’s available cognitive resources is shaped during task execution. In this respect, understanding the effects of AI use on learning requires a theoretical framework grounded in a mental workload perspective. Mental Workload Theory (MWT) posits that performance is contingent upon the relationship between the limited cognitive resources of the individual and the demands imposed by the task (Hart & Staveland, 1988; Wickens, 2008). This framework emphasizes that dimensions such as mental demand, time pressure, effort, and emotional strain experienced during task performance are closely related to perceived task performance. Accordingly, understanding AI-supported learning requires attention not only to workload itself, but also to how workload-related experiences are associated with learners’ perceptions of performance and task effectiveness. AI-supported tools can alter the demand structure of academic tasks by restructuring task complexity, accelerating information processing, and externalizing certain cognitive operations. From a cognitive offloading perspective, learners may use external tools to support cognitively demanding activities such as information searching, organizing ideas, and generating preliminary content, thereby redistributing cognitive effort across different stages of task completion (Risko & Gilbert, 2016). In this sense, AI-supported tools may function as external cognitive supports that influence how learners allocate their limited cognitive resources during academic tasks. Such changes may influence how individuals allocate their limited cognitive resources to meet task requirements, thereby leading to variations in perceived mental workload. Accordingly, examining how the frequency of AI use reshapes perceived workload during academic tasks constitutes a meaningful line of inquiry within the framework of mental workload theory.

According to mental workload theory, individuals allocate their limited cognitive resources across competing task demands during performance. When task demands exceed an individual’s cognitive capacity, a state of overload may occur, potentially leading to increased error rates, decreased performance, and heightened emotional strain. Conversely, when task demands fall substantially below one’s capacity, underload may emerge, which can result in reduced motivation and diminished attentional engagement. Optimal performance is achieved when there is a balanced alignment between task demands and available cognitive resources—an optimal level of workload (Wickens, 2008). Within this framework, dimensions such as mental demand, temporal demand, and effort are interpreted as indicators of cognitive resource expenditure, whereas frustration reflects the emotional consequences of a mismatch between demands and resources (Hart & Staveland, 1988). This perspective offers a useful analytical lens for examining task-based cognitive experiences in digital and AI-supported learning environments. By facilitating task structuring, accelerating access to information, and organizing procedural steps, AI tools may reduce perceived task demands and alleviate pressure on learners’ cognitive resources. At the same time, excessive reliance on AI may lower task demands to a degree that diminishes active cognitive engagement. In such cases, perceived performance may increase even as the depth of cognitive investment shifts, raising important questions about the nature of learning under AI-supported conditions. In this regard, a conceptual parallel can be drawn with cognitive load theory (CLT), which similarly underscores the importance of cognitive resource allocation in learning processes (Sweller, 2024; Sweller et al., 2011, 2019). However, MWT provides a more direct framework for analyzing subjective workload and its relationship to performance during AI-supported academic tasks. It is particularly suitable for the present study because it enables the examination of subjective, task-based workload dimensions—such as mental demand, temporal demand, effort, frustration, and perceived performance—that are directly aligned with the NASA-TLX framework used in this study.

In conclusion, the effects of AI use should not be evaluated solely in terms of performance outcomes, but also through the components of perceived mental workload experienced during task execution. Examining how the frequency of AI use reshapes dimensions such as mental demand, temporal pressure, effort, and emotional strain is essential for understanding how AI tools influence the balance between task demands and learners’ cognitive resources. From the perspective of mental workload theory, AI use is better conceptualized not as a unidirectional mechanism for reducing workload, but as a factor that regulates the alignment between cognitive resources and task demands. This view challenges the assumption that workload automatically decreases as AI use increases and instead raises the possibility that certain levels of use may foster a more balanced—and potentially optimal—workload structure. In this context, it is insufficient to characterize the impact of AI on learning merely in terms of “enhancing achievement” or “facilitating learning.” What is more consequential is how the frequency of AI use structures the components of mental workload and how these configurations relate to perceived task performance. Although prior research has examined the general effects of AI-supported systems on learning outcomes, studies that specifically address the differentiating role of usage frequency on mental workload dimensions—and its association with perceived performance, particularly among pre-service teachers—remain limited (Demiralay, 2025). For instance, Yanar and Ergene (2025) underscore the need for further investigation into the cognitive and performance-related implications of AI use in teacher education contexts. Moreover, existing studies have predominantly focused on AI adoption, acceptance, or perceived usefulness, with considerably less attention given to how varying levels of AI engagement relate to workload experiences during academic tasks. Accordingly, the present study sought to contribute to the literature by examining AI use frequency as a differentiating factor in pre-service teachers’ mental workload and perceived performance. From a mental workload theory perspective, the relationship between AI use frequency and perceived workload may not necessarily be linear. For pre-service teachers, limited AI use may be associated with higher workload because unfamiliar tools can add demands related to understanding, operating, and integrating AI-generated outputs into pedagogical design tasks. However, very frequent use may also introduce additional demands, such as monitoring AI-generated content, evaluating its pedagogical appropriateness, revising inaccurate or incomplete outputs, and deciding how much responsibility should remain with the teacher candidate. Therefore, moderate AI use may be associated with more favorable perceived workload profiles in some dimensions because it may provide support without fully shifting attention toward supervision and verification. This theoretical possibility suggests that AI use frequency should be examined not only in terms of linear differences, but also in terms of potential nonlinear patterns across NASA-TLX-based workload dimensions.

This study contributes theoretically by extending mental workload theory to AI-supported pedagogical design in pre-service teacher education. It frames AI use not merely as technology adoption, but as a workload-related experience shaped by task demands, perceived usefulness, and developing pedagogical judgment. By showing that AI use frequency is associated with dimension-specific and partly nonlinear workload patterns, the study suggests that lower perceived workload should not automatically be interpreted as educationally beneficial in teacher education. Thus, the study links AI use, perceived workload, and professional learning in a context where learners are still developing teaching competence. The aim of this study was to examine how pre-service teachers’ frequency of artificial intelligence tool use is associated with the mental workload they experience during assignment-related academic tasks and their perceived task performance. Within this framework, task-based workload components—such as mental demand, temporal demand, effort, and emotional strain—were investigated in relation to varying levels of AI usage frequency through the lens of mental workload theory. In doing so, the study sought to provide a more systematic account of how AI use restructures the balance between cognitive resources and task demands, and how this restructuring relates to perceptions of performance. Focusing on pre-service teachers adds pedagogical significance to the study, as these individuals are likely to use AI not only in their own learning processes but also in their future classroom practices. This issue is particularly important in teacher education because uncritical or poorly calibrated AI use may reduce the cognitive engagement through which pre-service teachers develop pedagogical reasoning, instructional decision-making, and professional judgment. For this reason, examining how AI use frequency is associated with perceived mental workload at this stage is particularly important. Because pre-service teachers are still developing instructional design skills and professional judgment, AI use was not expected to function simply as a workload-reducing tool. Rather, its association with workload was expected to depend on the extent to which candidates were familiar with AI tools and able to integrate them into task-related decision-making.

Accordingly, the study addressed the following research questions:

RQ1. What are pre-service teachers’ AI use profiles, purposes of AI use, and perceptions of the usefulness of AI tool functionalities in academic and teacher-preparation contexts?
RQ2. Do NASA-TLX-based perceived workload dimensions and perceived task performance differ according to AI use frequency?
RQ3. Does the association between AI use frequency and NASA-TLX-based perceived workload dimensions follow linear and/or nonlinear patterns?

## 2. Method

### 2.1. Research Design

This study was conducted within a quantitative research framework using a cross-sectional survey design, which is commonly employed to examine existing conditions, perceptions, and relationships among variables without manipulation (Creswell & Creswell, 2018).

### 2.2. Participants

The study sample consisted of 464 pre-service teachers enrolled in a faculty of education. Participants were recruited from professional knowledge courses in the faculty of education in which they were assigned an AI-supported pedagogical design task. Participants represented different stages of their teacher education programs: 217 (46.8%) were second-year students, 112 (24.1%) were in their third year, and 135 (29.1%) were fourth-year students. This distribution allowed the study to reflect perspectives from students with varying levels of academic and field experience. In terms of gender, the majority of participants were female (n = 369, 79.5%), while 95 (20.5%) identified as male. This distribution is consistent with the gender profile commonly observed in teacher education programs.

### 2.3. Data Collection

Data were collected in the first half of 2025 through an online structured questionnaire designed to examine pre-service teachers’ patterns of artificial intelligence use and their perceptions of AI tool functionalities in academic contexts (see Appendix A for the survey questionnaire). The instrument comprised four sections aligned with the research objectives. The first section assessed AI usage profiles, including frequency of use, variety of tools employed, and the specific AI platforms participants had experience with. This section aimed to capture both the intensity and scope of AI engagement. AI use frequency was assessed with reference to participants’ recent AI use in academic and teacher-preparation-related activities during the period in which they completed the AI-supported pedagogical design task. Thus, participants reported how often they used AI tools in relation to their recent coursework and task-related preparation within that semester. The second section focused on the purposes of AI use. Participants reported how often they used AI for different academic activities such as information seeking, research support, material preparation, presentations, and instructional content development, allowing differentiation between general academic support and pedagogically structured tasks. The third section examined the perceived usefulness of AI features. Participants evaluated the value of functionalities such as time saving, rapid feedback, idea generation, content support, language assistance, and accuracy-related guidance, providing insight into how AI is perceived as a productivity aid versus a reliable academic support tool.

In the final section, the NASA-TLX (Task Load Index) was used to assess participants’ post-task perceived workload associated with a specific AI-supported pedagogical design task. Developed by Hart and Staveland (1988) at NASA Ames Research Center, NASA-TLX is a widely used multidimensional subjective workload assessment tool that evaluates task load across six dimensions: mental demand, physical demand, temporal demand, perceived performance, effort, and frustration level. In this study, the raw TLX version was adapted to the teacher education context and administered after a specific course-based pedagogical design task. Within their professional knowledge courses, participants were assigned to prepare a lesson plan that included the design of an instructional activity and the development of teaching materials. After completing this task with the support of AI tools, participants responded to the NASA-TLX dimensions by evaluating the workload they experienced during this specific task, rather than their general academic workload. Participants rated each dimension on a scale from 0 (very low) to 100 (very high). To simplify the data collection process, the raw TLX version was employed in this study, without applying individual weightings to the dimensions. Because participants did not complete a single experimentally standardized task immediately before completing the questionnaire, the resulting scores should be interpreted as NASA-TLX-based retrospective perceived workload ratings for AI-supported pedagogical design tasks, rather than as task-specific NASA-TLX scores obtained during or immediately after a single defined task. The NASA-TLX has been extensively used across various domains, including education, healthcare, and aviation, and has been reported in previous research to have high internal consistency and construct validity. In the present study, its dimensions were used to capture perceived mental, temporal, emotional, and effort-related aspects of workload in AI-supported teacher-preparation tasks. The performance item was adapted to assess participants’ perceived success in completing the AI-supported lesson planning task. Unlike the original NASA-TLX performance scale, in which the rating is anchored from good performance to poor performance, the adapted item in this study was scored so that higher values indicated higher perceived task performance. Therefore, this item was reported as perceived task performance and interpreted separately from the workload-demand dimensions. No overall NASA-TLX workload score was computed by summing or averaging the six dimensions; thus, the adapted performance item was not combined with the other dimensions in a way that would require reverse coding.

### 2.4. Data Analysis

Data analysis was conducted using statistical procedures appropriate for the study’s descriptive and comparative aims. Prior to analysis, the dataset was screened for completeness and entry errors. Descriptive statistics, including frequencies, percentages, means, and standard deviations, were calculated to summarize pre-service teachers’ AI usage patterns, purposes of use, and perceived usefulness of AI tool features. These analyses provided an overall profile of how AI tools were integrated into participants’ academic practices.

To examine differences related to frequency of AI use, participants were grouped according to their reported usage levels. Before conducting inferential analyses, normality was assessed by examining skewness and kurtosis values for each usage group. For all three groups, skewness and kurtosis values fell within the acceptable range of −2 to +2, indicating no serious deviation from normality and supporting the use of parametric tests (George & Mallery, 2010). Group comparisons were then performed to determine whether patterns of AI engagement were associated with variations in perceived workload and performance-related perceptions. One-way analysis of variance (ANOVA) was used to test for statistically significant differences between usage groups. When significant effects were identified, post hoc comparisons were conducted to determine which specific group differences accounted for the overall effect. Effect sizes were also calculated to evaluate the practical significance of observed differences beyond statistical significance.

### 2.5. Findings

This section presents the results of the analyses conducted to address the research objectives. The findings are organized according to the main variables of the study, including patterns of AI use, purposes of use, perceived usefulness of AI tool features, and cognitive workload.

### 2.6. Profiles of Artificial Intelligence Use

The results reveal considerable heterogeneity in pre-service teachers’ engagement with artificial intelligence (AI) tools rather than a uniform pattern of use. The largest group of participants, representing 51.08% of the sample (n = 237), reported using AI tools “A few times a week,” while 19.40% (n = 90) reported daily use (“Every day”). At the same time, a substantial proportion of participants, 29.53% (n = 137), indicated that they use AI tools only “Rarely,” whereas no participants reported never using them. On a 4-point Likert scale (1 = Never, 4 = Every day), the overall mean usage frequency was 3.14 (SD = 0.73). Taken together, these findings suggest that AI use is present across the sample, but not ubiquitous: rather, pre-service teachers differ meaningfully in the extent to which they incorporate AI tools into their academic or professional routines. Regarding the diversity of tools used, most participants reported employing a limited range of AI applications. The majority indicated using between one and three different tools, with the overall mean number of tools used being 2.06 (SD = 1.06). This finding implies that while the integration of AI into educational practice is becoming more common, pre-service teachers tend to rely on a small set of familiar and accessible platforms rather than a wide array of specialized tools. When participants were asked to identify the specific AI tools they had used, ChatGPT (GPT-4o) emerged as the most frequently utilized platform, reported by 435 individuals. Other commonly mentioned tools included Canva AI (Magic Studio) (231), Gemini (2.0) (128), and Microsoft Copilot (72). In contrast, tools such as Grammarly were mentioned by only a few participants (14). These usage patterns reflect a strong preference for general-purpose generative AI platforms—particularly those that facilitate text generation, presentation design, and multimodal content creation—over domain-specific or pedagogically tailored AI applications.

### 2.7. Purposes of AI Use

**Figure 1:**
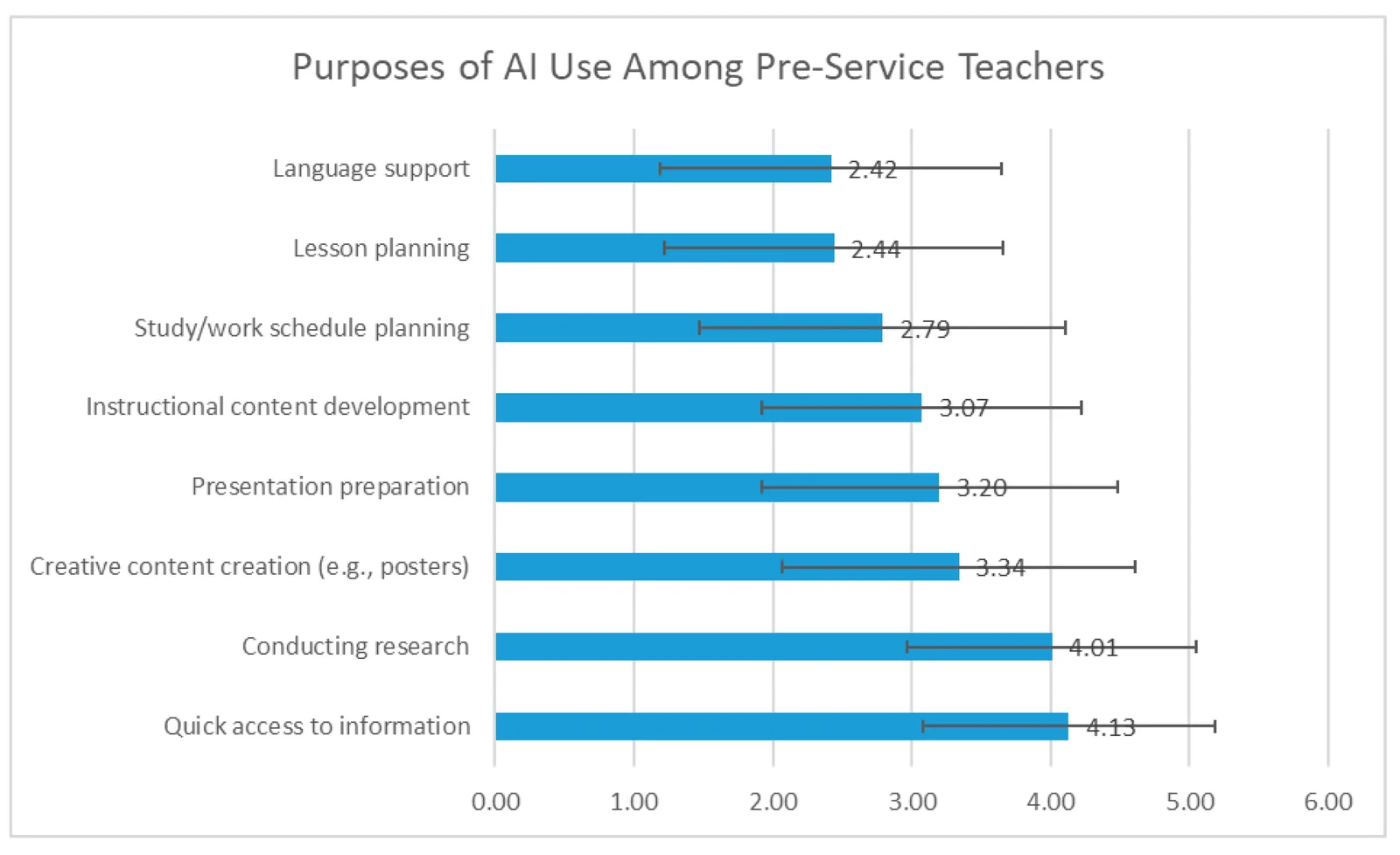
Distribution of AI tool use purposes reported by pre-service teachers. Note: Error bars represent ±1 standard deviation.

Figure 1 presents the AI use purposes of pre-service teachers.

Figure 1 presents the self-reported purposes for which pre-service teachers use AI tools. Participants rated how frequently they used AI tools for different academic and teacher-preparation-related tasks on a 5-point Likert scale (1 = Never, 5 = Always). The error bars in Figure 1 represent standard deviations, indicating variation in participants’ reported frequency of AI use across purposes. These results should be interpreted as pre-service teachers’ self-reported purposes of AI use in academic and teacher-preparation-related activities, rather than as direct evidence of changes in classroom instruction. The highest mean scores were observed for gaining quick access to information (M = 4.13, SD = 1.05) and conducting academic research (M = 4.01, SD = 1.04). These findings suggest that among pre-service teachers, AI use is most strongly associated with information-oriented activities, such as locating, organizing, or initially exploring academic content. However, these categories should be interpreted cautiously, as they may reflect AI-supported information seeking rather than direct integration of AI into teaching practice.

Moderate levels of AI use were reported for creative and semi-structured academic tasks, including creating visual materials such as posters or graphics (M = 3.34, SD = 1.27) and preparing academic presentations (M = 3.20, SD = 1.28). These results indicate that some pre-service teachers use AI tools to support the production and presentation of academic materials in their coursework or teacher education activities. By contrast, relatively lower levels of use were reported for tasks more directly related to pedagogical planning and instructional preparation. For example, using AI for instructional content development yielded a mean of 3.07 (SD = 1.15), while planning study or work schedules was rated lower (M = 2.79, SD = 1.32). The least frequently reported purposes were language support (M = 2.42, SD = 1.23) and lesson planning (M = 2.44, SD = 1.22). Overall, the findings suggest that pre-service teachers’ AI use is more strongly concentrated in information access and academic support activities than in pedagogical planning tasks such as lesson planning or instructional content development.

### 2.8. Perceived Usefulness of AI Tool Features

**Figure 2:**
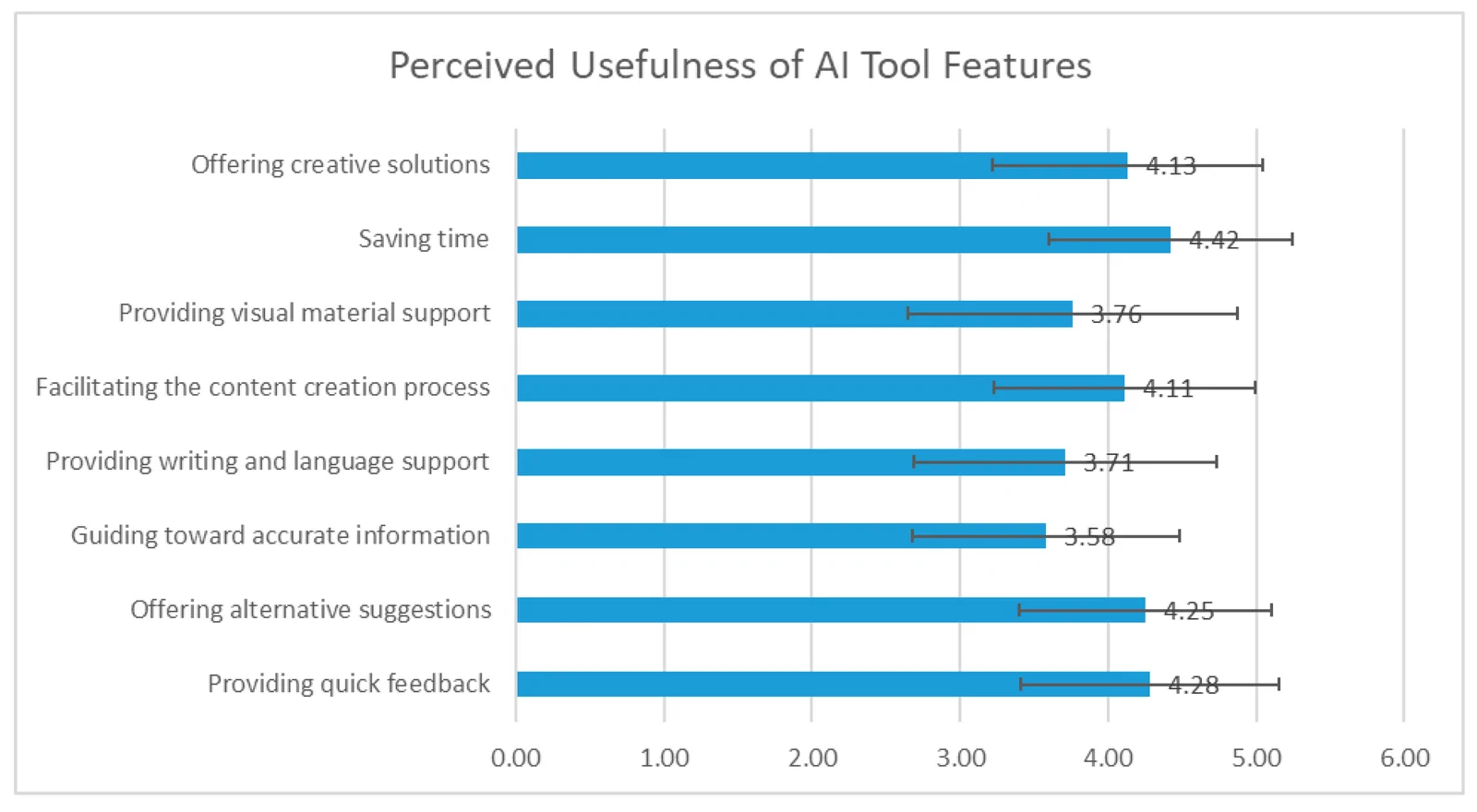
Perceived usefulness of ai tool features: mean ratings across key functionalities. Note: Error bars represent ±1 standard deviation.

Figure 2 presents the perceived usefulness of AI tools according to the pre-service teachers.

Participants evaluated the perceived usefulness of various features offered by artificial intelligence tools using a 5-point Likert scale (1 = Not useful at all, 5 = Extremely useful). The results revealed that the most highly rated feature was time saving, with a mean score of 4.42 (SD = 0.82), reflecting participants’ strong appreciation for the efficiency benefits provided by AI systems. This was followed by fast feedback (M = 4.28, SD = 0.87) and alternative suggestions (M = 4.25, SD = 0.85), both of which further emphasize AI’s role in accelerating decision-making and enhancing productivity. Other features perceived as notably beneficial included creative solution generation (M = 4.13, SD = 0.91) and support for content creation (M = 4.11, SD = 0.88), indicating that AI is particularly valued for its capacity to inspire ideas and assist in the construction of academic or instructional materials. Although rated slightly lower, the remaining features were still viewed positively. Visual material support received a mean score of 3.76 (SD = 1.11), while language support was rated at 3.71 (SD = 1.02). The feature with the lowest perceived usefulness was accurate guidance, with a mean score of 3.58 (SD = 0.90), suggesting that some participants may be skeptical about AI’s ability to provide consistently precise or contextually appropriate feedback.

### 2.9. Mental Workload (NASA-TLX) Results

**Figure 3:**
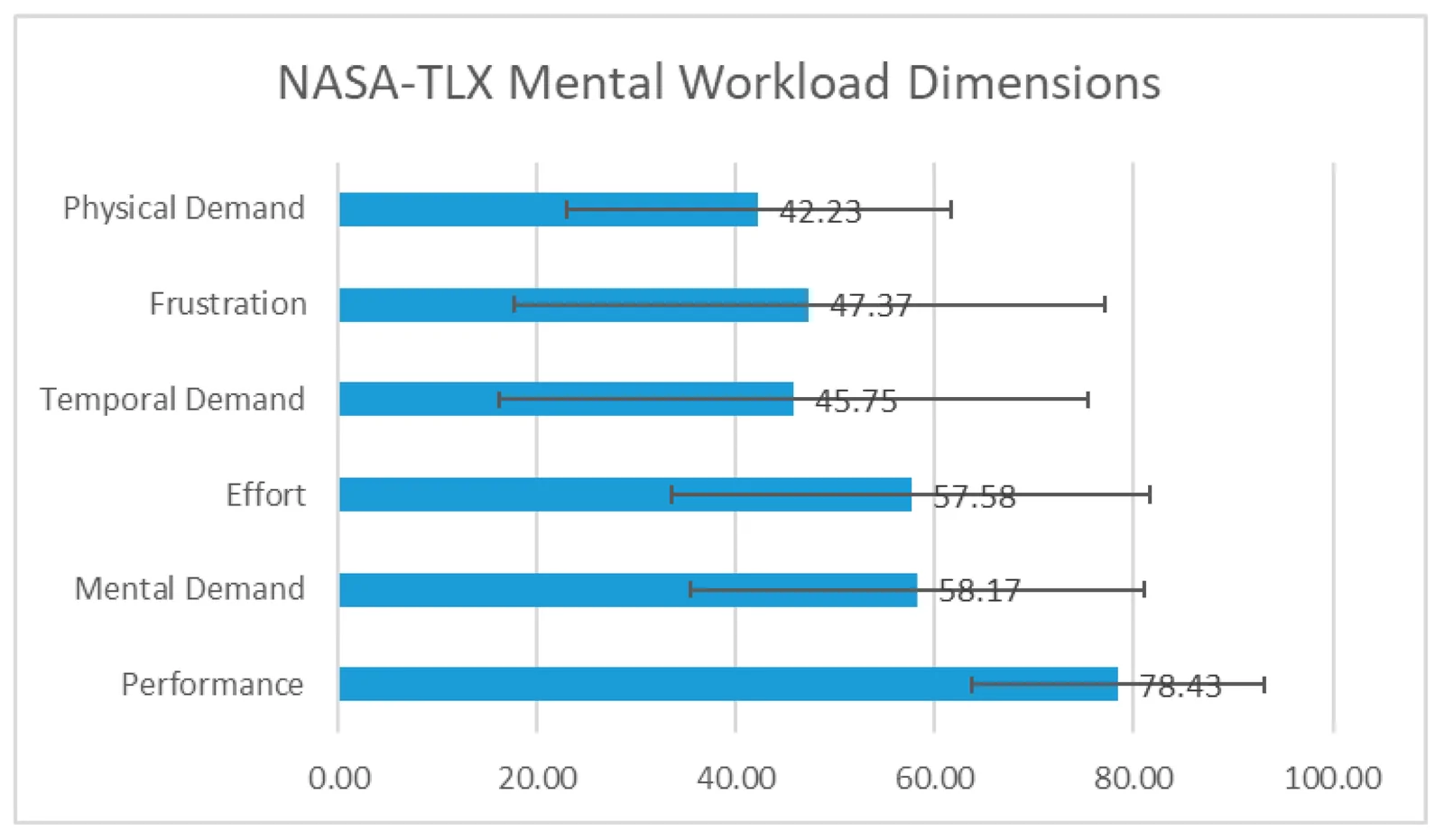
Mean scores on NASA-TLX mental workload dimensions. Note: Error bars represent ±1 standard deviation.

Participants’ perceived mental workload during task performance was assessed using the NASA Task Load Index (NASA-TLX), which includes six dimensions: mental demand, physical demand, temporal demand, perceived task performance, effort, and frustration. Figure 3 presents the visualization of the mean scores of mental workload.

The highest mean score was observed for perceived task performance (M = 78.43, SD = 14.67), indicating that participants generally rated their success in completing the AI-supported lesson planning task positively. Contrary to initial expectations, mental demand (M = 58.17, SD = 22.80) and effort (M = 57.58, SD = 24.00) yielded moderately high scores, suggesting that while AI tools may assist in reducing workload, a substantial amount of cognitive and physical engagement remains necessary. Temporal demand was rated at a moderate level (M = 45.75, SD = 29.68), indicating that pre-service teachers occasionally experienced time-related pressure during academic activities. Similarly, the frustration level (M = 47.37, SD = 29.64) suggested the presence of emotional strain, albeit to a lesser extent. As anticipated, physical demand received the lowest average score (M = 42.23, SD = 19.29), which is consistent with the predominantly cognitive nature of academic work.

### 2.10. Mental Workload Differences by AI Usage Frequency

The mental demand dimension showed notable variation across groups. Interestingly, the highest mental demand was reported by those who rarely used AI tools (M = 76.60), while those using AI daily reported a considerably lower level (M = 61.16), and the lowest was observed among occasional users (M = 46.39). This trend suggests that regular AI users may experience reduced cognitive strain, potentially due to improved efficiency and task structuring facilitated by AI. Temporal demand followed a similar pattern, with rare users reporting the highest perceived time pressure (M = 75.04), in contrast to lower values among daily (M = 35.51) and occasional users (M = 32.72). This indicates that AI usage may contribute to more effective time management during academic tasks. Regarding physical demand, rare users again reported the highest levels (M = 47.64), while occasional users indicated the lowest (M = 37.62). Although academic work is primarily cognitive, these findings imply that AI support may reduce perceived manual or procedural effort.

Effort scores were also highest among rare users (M = 76.34), followed by daily users (M = 62.68), and lowest in the occasional users (M = 44.81), reinforcing the idea that more frequent interaction with AI tools correlates with reduced perceived workload. When evaluating frustration, participants who rarely used AI tools reported significantly higher emotional strain (M = 70.26), compared to occasional users (M = 41.11) and especially daily users (M = 28.47). This may suggest that limited familiarity or efficiency with AI tools can lead to greater emotional tension during task execution. Lastly, perceived task performance scores were highest among daily users (M = 86.72) and occasional users (M = 79.69), whereas rare users rated their perceived task performance lowest (M = 70.82). This indicates that frequent AI usage is associated with a greater sense of achievement and competence in academic contexts.

**Figure 4:**
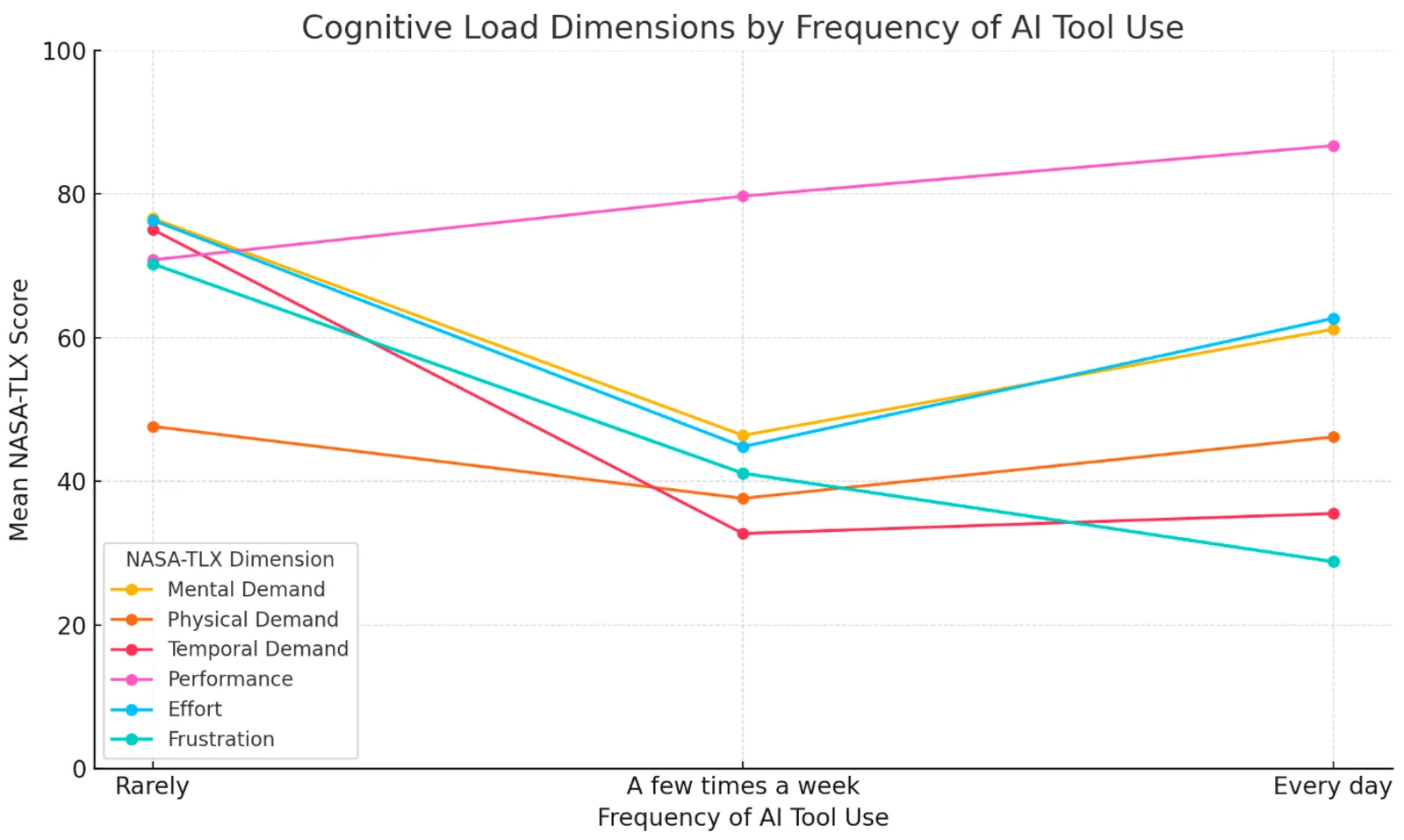
Changes in mental workload dimensions by frequency of AI tool use (NASA-TLX scores).

Figure 4 illustrates how different mental workload dimensions, as measured by the NASA-TLX scale, vary according to the frequency of AI tool use among pre-service teachers. A clear trend emerges in which participants who report rarely using AI tools experience higher levels of mental demand, effort, temporal demand, and frustration, compared to those who use such tools more frequently. Specifically, mental and temporal demands sharply decrease as AI usage increases, suggesting that regular engagement with AI tools helps offload cognitive and time-related pressures in academic tasks. Interestingly, the perceived task performance scores improve steadily across usage levels, with the highest self-perceived performance reported by those using AI tools daily. This implies that frequent users not only experience reduced workload but also feel more competent in meeting academic objectives. Meanwhile, physical demand remains the lowest across all groups, which is consistent with the predominantly cognitive nature of the tasks assessed.

The results of the one-way analysis of variance (ANOVA) conducted to determine whether NASA-TLX perceived task load dimension scores differ according to the frequency of AI tool use are presented in Table 1.

As shown in Table 1, the mean scores of all NASA-TLX mental workload subscales differed significantly according to the frequency of AI tool use: mental demand (*F*(2, 461) = 115.13, *p* < 0.001, η^2^ = 0.33), physical demand (*F*(2, 461) = 14.88, *p* < 0.001, η^2^ = 0.06), temporal demand (*F*(2, 461) = 160.04, *p* < 0.001, η^2^ = 0.41), performance (*F*(2, 461) = 39.25, *p* < 0.001, η^2^ = 0.15), effort (*F*(2, 461) = 115.97, *p* < 0.001, η^2^ = 0.33), and frustration (*F*(2, 461) = 88.42, *p* < 0.001, η^2^ = 0.28). According to Cohen’s (1988) benchmarks, eta-squared values of 0.01, 0.06, and 0.14 may be interpreted as small, medium, and large effects, respectively. Thus, differences across AI tool use-frequency groups were large for all dimensions except physical demand, which showed a medium-sized effect. These findings suggest that AI tool use frequency is associated with perceived task load and task performance among pre-service teachers. However, the pattern was not uniformly linear: occasional users reported the lowest mental demand and effort, whereas daily users reported the highest perceived task performance and the lowest frustration.

To further examine which specific group comparisons contributed to these significant differences, a post hoc analysis was conducted following the ANOVA. Post hoc analyses conducted following ANOVA, taking into account the homogeneity of variances assessed by Levene’s test, revealed that all group comparisons were statistically significant (*p* < 0.05) across the mental workload dimensions—except in two specific cases. No statistically significant difference was found between rare and daily users in the physical demand dimension, and between occasional and daily users in the temporal demand dimension. All other pairwise comparisons were statistically significant at *p* < 0.05. These exceptions aside, the results demonstrate that the frequency of AI tool use significantly influences perceived mental workload, with meaningful differences observed across most user groups. Post hoc comparisons based on Cohen’s *d* revealed the following group-level effect sizes across NASA-TLX dimensions. Table 2 presents pairwise Cohen’s *d* effect sizes for differences in NASA-TLX mental workload subscales across AI usage frequency groups (rare, occasional, daily). Effect sizes are interpreted according to Cohen’s (1988) guidelines: *d* = 0.2 (small), *d* = 0.5 (medium), *d* = 0.8 (large).

Post hoc tests identified statistically significant pairwise differences, and Cohen’s d values were used to evaluate the magnitude of these differences. The analysis demonstrated that pre-service teachers who used AI tools more frequently (occasionally or daily) consistently reported lower levels of mental workload in comparison to rare users, particularly in the domains of mental demand, effort, and frustration. The most substantial effect sizes were observed between rare and occasional users, especially in mental demand (d = 1.55), effort (d = 1.54), and frustration (d = 1.12), all of which exceed Cohen’s (1988) conventional benchmark for a large effect. Additionally, comparisons between rare and daily users yielded large effects in mental demand (d = 0.88), effort (d = 0.83), and frustration (d = 1.70), indicating that daily users reported lower mental demand, effort, and frustration than rare users. Interestingly, in some cases (e.g., mental demand, d = –0.80), occasional users reported even lower load than daily users, suggesting that the most favorable workload profile was observed among occasional users rather than daily users for some dimensions.

Because AI use frequency consisted of three ordered categories, polynomial trend analyses were conducted to examine whether the observed group differences reflected linear and/or quadratic patterns. Given indications that occasional users sometimes reported more favorable workload outcomes than daily users, a curvilinear association was theoretically plausible. To examine whether the relationship between AI use frequency and perceived workload dimensions was nonlinear, hierarchical polynomial regression analyses were conducted for each NASA-TLX dimension. In all analyses, the centered AI use-frequency variable (Xc) was entered in Step 1, followed by the quadratic term (Xc^2^) in Step 2. AI use frequency was represented by three ordered categories: rare use, occasional use, and daily use. With three ordered groups, the full quadratic model is mathematically equivalent to the one-way ANOVA model in terms of explaining between-group differences; therefore, the regression results were interpreted as polynomial trend components rather than as independent evidence of additional group differences.

For the mental demand dimension, the linear model was significant (*R*^2^ = 0.095, *F*(1, 462) = 48.55, *p* < 0.001). Adding the quadratic term significantly improved the model (Δ*R*^2^ = 0.238, Δ*F*(1, 461) = 164.53, *p* < 0.001), and the full quadratic model explained 33.3% of the variance (R^2^ = 0.333). The quadratic coefficient was positive and significant (B = 22.49, *p* < 0.001), indicating a nonmonotonic pattern across AI use-frequency groups. Consistent with the group means, mental demand was highest among rare users (M = 76.59), lowest among occasional users (M = 46.39), and higher again among daily users (M = 61.15). Regarding the physical demand subdimension, the linear model was not significant (*R*^2^ = 0.004, F(1, 462) = 1.81, *p* > .05). However, adding the quadratic term significantly improved model fit (Δ*R*^2^ = 0.057, Δ*F*(1, 461) = 27.86, *p* < 0.001), and the full quadratic model explained 6.1% of the variance (*R*^2^ = 0.061). The quadratic term was significant (B = 9.29, *p* < 0.001), suggesting a modest nonmonotonic pattern, with physical demand lower among occasional users (M = 37.61) than among rare users (M = 47.64) and daily users (M = 46.16). The linear model explained a substantial proportion of variance for the temporal demand (*R*^2^ = 0.268, *F*(1, 462) = 169.53, *p* < 0.001). The inclusion of the quadratic term significantly improved model fit (Δ*R*^2^ = 0.142, Δ*F*(1, 461) = 110.42, *p* < 0.001), and the full quadratic model explained 41.0% of the variance (*R*^2^ = 0.410). The positive quadratic coefficient (B = 22.56, *p* < 0.001) indicated a nonlinear pattern across the three groups. Temporal demand was highest among rare users (M = 75.03), lowest among occasional users (M = 32.71), and only slightly higher among daily users (M = 35.51). Therefore, this pattern should be interpreted as group-specific nonlinearity rather than strong evidence of an optimal usage level.

For the effort dimension, the linear model was significant (*R*^2^ = 0.075, *F*(1, 462) = 37.60, *p* < 0.001). Adding the quadratic term substantially increased explained variance (Δ*R*^2^ = 0.260, Δ*F*(1, 461) = 179.81, *p* < 0.001), and the full quadratic model explained 33.5% of the variance, (*R*^2^ = 0.335). The quadratic coefficient was strongly positive (B = 24.71, *p* < 0.001), showing a nonmonotonic association. Effort was highest among rare users (M = 76.34), lowest among occasional users (M = 44.80), and higher again among daily users (M = 62.67). The linear model was also significant for frustration (*R*^2^ = 0.258, *F*(1, 462) = 160.90, *p* < 0.001). The quadratic model provided a small but significant improvement (Δ*R*^2^ = 0.019, Δ*F*(1, 461) = 12.09, *p* < 0.001), and the full quadratic model explained 27.7% of the variance (*R*^2^ = 0.277). The quadratic term (B = 8.26, *p* = .001) indicated a small departure from strict linearity. However, the descriptive means did not support a U-shaped interpretation for frustration. Instead, frustration decreased across the three groups, from rare users (M = 70.25) to occasional users (M = 41.10) and daily users (M = 28.47). For perceived task performance, the linear model was significant (*R*^2^ = 0.145, *F*(1, 462) = 78.07, *p* < 0.001). However, the quadratic term did not significantly improve the model (Δ*R*^2^ = 0.001, ΔF(1, 461) = 0.51, *p* = .473). The full quadratic model explained approximately 14.6% of the variance (R^2^ = 0.146). Thus, perceived task performance was best characterized by a linear trend rather than a quadratic pattern. Descriptive means showed that perceived task performance increased across AI use-frequency groups, from rare users (M = 70.81) to occasional users (M = 79.68) and daily users (M = 86.72).

Across the NASA-TLX-based dimensions, the results revealed dimension-specific patterns rather than a consistent U-shaped relationship between AI use frequency and perceived workload. Mental demand, physical demand, temporal demand, and effort showed lower scores among participants who used AI occasionally than among rare users, although the extent to which scores increased again among daily users varied by dimension. Frustration, in contrast, decreased across AI use-frequency groups, while perceived task performance increased with AI use frequency. These findings suggest that the association between AI use frequency and NASA-TLX dimensions varies across workload components. Some dimensions, particularly mental demand and effort, showed nonmonotonic patterns, whereas frustration decreased and perceived task performance increased as AI use frequency increased.

## 3. Discussion

The present study sought to understand not only whether pre-service teachers use artificial intelligence tools, but how such use is positioned within their academic practices and how it is associated with perceived workload and perceived task performance in AI-supported pedagogical design tasks. Rather than treating AI adoption as a purely technical matter, the findings suggest that AI is becoming part of pre-service teachers’ academic and teacher-preparation practices, shaped by perceived usefulness and task-related workload experiences. Examining patterns of use, perceived benefits, and mental workload together provides a more comprehensive picture of how AI is being incorporated into pre-service teachers’ academic experiences and where tensions or limitations in this integration may lie. In this respect, the study contributes to the literature by positioning AI use in pre-service teacher education as a perceived workload issue, not only as a question of adoption, usefulness, or digital competence. Within this sample, the findings suggest that artificial intelligence tools were reported as a common component of pre-service teachers’ digital practices rather than as entirely peripheral technologies. However, the observed variation in engagement levels suggests that AI use should not be interpreted as a direct indicator of professional competence: instead, engagement with AI appears to be associated with individual differences in experience, perceived usefulness, and perceived pedagogical relevance. The pattern observed in this study is consistent with the broader normalization of AI-enabled tools across higher education, where adoption is expanding faster than the development of shared pedagogical norms and educator-facing guidance (Zawacki-Richter et al., 2019). At the same time, the variation in uptake across individuals is theoretically unsurprising: research on technology acceptance among pre-service teachers repeatedly shows that intentions and actual use tend to differ as a function of perceived usefulness/ease of use, facilitating conditions, and trust—factors that can create uneven engagement even within the same program context (Sun et al., 2025; Teo, 2010). From a teacher-education perspective, this combination suggests a transition phase in which AI use is becoming “ordinary,” yet remains unevenly internalized as a professional practice. Accordingly, capacity-building efforts should not assume uniform readiness, but rather foreground critical AI literacy and responsible-use competencies alongside access and skill development (UNESCO, 2023).

This uneven internalization may also be related to shape how AI is functionally positioned within academic work, as reflected in the purposes for which these tools are used. Participants’ self-reports suggest that AI was used chiefly for rapid access to information and for supporting academic inquiry and preparation processes. In contrast, its use seems more limited in tasks that require structured pedagogical decision-making, suggesting a clearer boundary between academic support and direct instructional design. This orientation may be interpreted as consistent with emerging discussions that frame generative AI as a possible form of cognitive offloading, where learners may rely on external digital systems for effortful processes such as searching, organizing, and initial drafting (Risko & Gilbert, 2016). In higher-education contexts, large language model–based tools are increasingly described as supports for idea generation, literature orientation, and early-stage knowledge construction rather than as autonomous instructional agents, which parallels the tendency to rely on AI for exploratory and preparatory tasks over formally structured pedagogical design (Kasneci et al., 2023; Zawacki-Richter et al., 2019). From a teacher-education perspective, this pattern suggests that pre-service teachers may be using AI to support academic problem-solving activities, yet still draw a boundary between assistance with learning-related processes and responsibility for core instructional planning (Açıkyıldız & Şahin, 2025; Asio & Gadia, 2024; Kasneci et al., 2023).

Participants’ self-reports indicate that they valued AI tools primarily for efficiency, speed, rapid feedback, and idea generation, while showing more caution toward features related to accuracy and linguistic precision. This pattern suggests that participants tended to perceive AI as a process-support and productivity-enhancing tool rather than as a fully reliable source for pedagogically sensitive decisions. This emphasis is consistent with the perceived usefulness dimension of technology acceptance research, which identifies performance gains and task facilitation as central drivers of positive attitudes toward new technologies (Bilgin & Güngören, 2025; Davis, 1989). In educational contexts, generative AI is similarly characterized as supporting brainstorming, drafting, and creative exploration, reinforcing its role as a tool that augments human ideation rather than replaces pedagogical judgment (Kasneci et al., 2023). Conversely, the more cautious stance toward features related to accuracy and linguistic precision reflects a pattern of calibrated trust often discussed in human–automation interaction research, where users differentiate between functions that enhance efficiency and those requiring high reliability and contextual sensitivity (Lee & See, 2004). Together, these tendencies suggest that pre-service teachers may view AI as a process-support technology while remaining cautious about relying on it for accuracy-sensitive and pedagogically consequential decisions.

Beyond perceived usefulness, the NASA-TLX-based workload findings provide a more nuanced picture of participants’ perceived workload during AI-supported pedagogical design work. The findings suggest that completing the AI-supported lesson planning task was associated with moderate levels of mental demand, effort, temporal demand, and frustration, whereas physical demand was comparatively low. At the same time, participants reported relatively high perceived task performance, suggesting that they generally evaluated themselves as successful in developing a lesson plan that included an instructional activity and teaching materials. This pattern is consistent with the possibility that AI tools are perceived as helpful in pedagogical design tasks, but the findings do not indicate that AI use eliminates the cognitive, temporal, and emotional demands of such work. One possible explanation is that even when AI is used, learners may still need to evaluate, adapt, organize, and integrate AI-generated content into pedagogically meaningful outputs. From a mental workload perspective, the findings may suggest that AI use is associated with lower perceived task-related burden for some users, while complex instructional design tasks may still require active cognitive processing, decision-making, and regulation of working memory resources (Sweller, 2010). At the same time, the persistence of notable mental demand and effort aligns with the view that digital tools may change how users experience cognitive demands rather than simply eliminating them, requiring users to engage in monitoring, evaluating, and integrating system outputs, which are themselves cognitively demanding activities (Paas & van Merriënboer, 2020). Moderate levels of temporal pressure and frustration further suggest that AI-supported environments may introduce new forms of process-related strain, consistent with research showing that interacting with intelligent systems involves cycles of interpretation, verification, and adjustment that carry both cognitive and affective costs (Longo, 2015). Taken together, these findings suggest that AI should not be interpreted as a simple load-reducing mechanism. Rather, AI-supported task completion may involve additional perceived demands related to evaluating and using AI-generated outputs, a possibility that should be examined directly in future research. Moreover, the pre-service teacher context is important for interpreting these workload-related findings. Unlike experienced teachers, pre-service teachers are still developing pedagogical reasoning, instructional design skills, and professional judgment. Therefore, lower perceived workload should not automatically be interpreted as an educationally desirable outcome. AI-supported reductions in perceived workload may be useful when they reduce unnecessary procedural demands, but they may be problematic if they reduce candidates’ engagement in pedagogical reasoning, critical evaluation, and independent instructional decision-making.

Importantly, mental workload patterns differed depending on how frequently AI tools were used. Participants who reported using AI tools infrequently experienced higher levels of mental demand, effort, time pressure, and frustration, whereas those with more regular use reported comparatively lower workload and stronger performance perceptions. This pattern suggests an association between AI use frequency and how participants perceived the demands of the academic task. One possible interpretation is that AI may be perceived as more useful or less demanding when learners have greater familiarity and routine in working with such tools; however, familiarity and routine were not directly measured in the present study. Higher reported mental demand, effort, time pressure, and frustration among infrequent users may be consistent with the idea that interacting with relatively unfamiliar technologies can impose additional perceived task-related demands unrelated to core academic processing, as learners must allocate attention both to the task itself and to understanding how to operate and interpret the system (Sweller, 2010). From a self-regulated learning perspective, more experienced users are likely better able to integrate external tools into their task strategies, coordinating planning, monitoring, and evaluation processes more efficiently, which may help explain why these participants reported lower perceived workload and higher perceived task performance (Panadero, 2017). In this sense, AI use should not be assumed to automatically reduce perceived workload: rather, its load-regulating potential appears to depend on users’ ability to embed it into effective task routines, echoing broader arguments that the educational value of digital tools is mediated by learners’ strategic and metacognitive engagement.

However, the relationship between AI use and workload was not strictly linear. Findings suggest that mental workload does not decrease in a strictly linear manner as AI use becomes more frequent. In several dimensions, participants who used AI tools at a moderate level reported more favorable workload profiles than those who used them daily, suggesting that the association between usage intensity and perceived workload is not purely incremental. This aligns with research suggesting that digital supports can both reduce and introduce cognitive load, depending on how they are integrated into task processes (Paas & van Merriënboer, 2020). While AI can streamline idea generation and task initiation, sustained or intensive use may require ongoing monitoring, evaluation, and correction of system outputs—activities that shift effort toward higher-order regulation rather than eliminating it. Such dynamics are consistent with the view that tools designed to enhance performance often redistribute cognitive effort toward supervision and quality control, especially when system outputs are not fully reliable (Lee & See, 2004). The observed pattern for some dimensions of the task loads may also be conceptually consistent with nonlinear models of performance, such as the Yerkes–Dodson principle, which proposes that optimal performance emerges at moderate rather than extreme levels of activation (Yerkes & Dodson, 1908). Although arousal was not directly measured in the present study, the finding that moderate AI use was associated with more balanced workload profiles suggests a possible nonmonotonic association between AI use frequency and some perceived workload dimensions, but they do not establish an optimal level of AI use. From a mental workload theory perspective, both overload (excessive demand) and underload (insufficient cognitive engagement) can undermine effective task performance (Wickens, 2008). Moderate AI use may therefore provide sufficient support to reduce unnecessary strain while preserving active cognitive engagement, whereas intensive use may shift effort toward continuous supervision and verification processes. Accordingly, the benefits of AI appear to depend less on constant exposure and more on developing calibrated, strategic use, reinforcing the need for teacher education to emphasize effective orchestration of AI within learning tasks rather than frequency of use alone. In this sense, AI literacy in teacher education must extend beyond access and familiarity to include regulation, evaluation, and strategic orchestration of AI within learning processes.

### 3.1. Implications for Practice and Research

These findings suggest that making AI tools available is only a first step: meaningful integration depends on how thoughtfully they are used. Although AI has become part of many pre-service teachers’ routines, its perceived value and workload-related implications appear to depend less on frequency alone and more on how well AI use is aligned with pedagogical purposes. Teacher education programs should therefore focus on developing practical AI literacy by helping future teachers learn how to evaluate AI-generated outputs, use prompts effectively, recognize the limitations of AI tools, and make ethically and instructionally sound decisions. The dimension-specific nonlinear patterns observed in some NASA-TLX workload dimensions also suggest that more frequent use is not necessarily more beneficial. Without structure, frequent reliance on AI may introduce additional demands related to monitoring, verifying, and revising AI-generated outputs. Guided practice in applying AI to lesson planning, assessment, instructional material development, and learning support may help pre-service teachers move from convenience-based use toward more pedagogically grounded applications.

The findings further suggest that AI-supported work does not necessarily remove cognitive or emotional demands. Concerns about task performance, effort, and occasional frustration may remain part of the experience, highlighting the importance of metacognitive and evaluative skills. Teacher education should therefore help pre-service teachers develop strategies for verifying AI-generated information, managing perceived workload, and using AI in a reflective and controlled way rather than treating it as an automatic solution. In this respect, AI literacy should include not only technical familiarity with AI tools but also critical judgment, pedagogical alignment, ethical awareness, and the ability to decide when AI support is or is not appropriate for a given teaching-related task.

For teacher education, the goal should not be to use AI simply to make pedagogical design tasks easier or faster. Rather, AI should be integrated in ways that preserve productive cognitive engagement while reducing unnecessary procedural burden. Teacher educators should therefore design AI-supported activities that require pre-service teachers to justify instructional choices, critique AI-generated suggestions, revise lesson plans based on pedagogical principles, and reflect on the limitations of AI outputs. Such practices may help ensure that AI supports professional learning rather than replacing the demanding forms of reasoning through which teaching competence develops.

For future research, it is important to look beyond whether AI is used and instead examine how it is used in real learning and teaching processes. Studies that explore interaction patterns, decision-making processes, prompt practices, verification strategies, and the development of AI-related competencies over time would provide deeper insight into effective integration. Research situated in authentic instructional contexts, such as lesson design, differentiation, assessment, and instructional material development, will be especially valuable for understanding whether AI becomes a tool for pedagogical transformation or remains mainly a productivity aid. Future studies should also combine self-report measures with objective indicators, such as task quality, achievement scores, accuracy, completion time, system logs, or learning analytics, to distinguish perceived workload and perceived task performance from actual learning outcomes.

### 3.2. Limitations and Future Research

Several limitations should be acknowledged. First, the study relied exclusively on self-report data. Therefore, the findings reflect participants’ subjective perceptions rather than objective indicators of workload, AI use, task performance, or learning success. In particular, the NASA-TLX performance dimension should be interpreted as perceived task performance, not as an objective measure of academic achievement or actual learning outcomes. Future studies should combine subjective workload measures with objective indicators such as task scores, accuracy, completion time, behavioral trace data, learning assessments, or system log data.

Second, the cross-sectional design limits the extent to which causal conclusions can be drawn. Although AI use frequency was significantly associated with several NASA-TLX dimensions, the findings do not show that AI use directly reduces workload or improves performance perceptions. It is also possible that students who already experience lower workload or higher confidence are more likely to use AI tools more frequently. Longitudinal and experimental studies are needed to clarify the direction and mechanisms of these relationships.

Third, AI use frequency was measured through broad self-reported categories. Although these categories allowed comparison between rare, occasional, and daily users, they do not capture the quality, duration, intensity, or pedagogical purpose of AI use. Future research should examine specific AI use practices and functions in greater detail.

Fourth, the interpretation of nonlinear patterns should be approached with caution. Because AI use frequency consisted of three ordered categories, the polynomial analyses should be understood as trend decompositions of group differences rather than as evidence of a continuous curvilinear relationship. Although some NASA-TLX dimensions showed nonmonotonic patterns, these results should be replicated with more fine-grained and continuous measures of AI use frequency before drawing strong conclusions about optimal levels of AI engagement.

Fifth, the sample was drawn from a single faculty of education and consisted predominantly of female pre-service teachers. This limits the generalizability of the findings to other teacher education programs, academic disciplines, institutional contexts, and more gender-balanced samples. In addition, the study was conducted within a single national context, and cultural, institutional, curricular, and policy-related factors may shape both AI tool use and perceived workload. Future studies should therefore include larger and more diverse samples across multiple institutions and national settings.

Finally, the present study focused on perceived workload and perceived task performance in relation to AI use frequency, but it did not directly examine the cognitive, motivational, or metacognitive mechanisms that may explain these associations. Future research could investigate how AI literacy, trust in AI, prompt-writing skills, self-regulated learning, verification strategies, and critical evaluation of AI-generated outputs influence the relationship between AI engagement and perceived workload.

## Figures and Tables

**Table 1:** One-way ANOVA results for NASA-TLX perceived task load dimensions by frequency of AI tool use (n = 464).

NASA-TLX Dimension	Rare Use (n = 137) M (SD)	Occasional Use (n = 237) M (SD)	Daily Use (n = 90) M (SD)	F	*p*	η^2^
Mental demand	76.59 (19.17)	46.39 (19.64)	61.15 (14.78)	115.13	<0.001	0.33
Physical demand	47.64 (19.60)	37.61 (19.54)	46.16 (14.76)	14.88	<0.001	0.06
Temporal demand	75.03 (22.14)	32.71 (22.99)	35.51 (23.53)	160.04	<0.001	0.41
Performance	70.81(18.98)	79.68 (11.53)	86.72 (7.33)	39.25	<0.001	0.15
Effort	76.34 (17.05)	44.80 (22.24)	62.67 (15.36)	115.97	<0.001	0.33
Frustration	70.25 (26.26)	41.10 (26.05)	28.47 (21.46)	88.42	<0.001	0.28

**Table 2:** Pairwise effect sizes (Cohen’s d) for NASA-TLX mental workload dimensions by AI usage frequency.

Dimension	Rare vs. Occasional	Rare vs. Daily	Occasional vs. Daily
Mental Demand	1.55 *	0.88 *	−0.80 *
Physical Demand	0.51 *	0.08	−0.47 *
Temporal Demand	1.86 *	1.74 *	−0.12
Performance	−0.6 *	−1.03 *	−0.67 *
Effort	1.54 *	0.83 *	−0.87 *
Frustration	1.12 *	1.70 *	0.50 *

Note. Positive d values indicate higher scores in the first group compared with the second group. Asterisks indicate statistically significant post hoc comparisons at *p* < 0.05.

## Data Availability

The datasets generated during and/or analyzed during the current study are available from the corresponding author on reasonable request.

## Appendix A. Survey Questionnaire on AI Use and NASA-TLX-Based Perceived Workload

Section 1. AI Use Profile
  1. How often do you use AI-supported tools? Please select one option.
    - I do not use them
    - Rarely
    - A few times a week
    - Every day
  2. Which AI applications do you use? Please list the AI applications or platforms you have used.
Section 2. Purposes of AI Use
  3. For what purposes do you use AI tools? Please rate each item on a scale from 1 to 5.
    (1 = I do not use them for this purpose to 5 = I use them very frequently for this purpose)
      - Preparing lesson plans
      - Developing content, such as instructional materials
      - Improving language, such as language support, grammar, or writing assistance
      - Conducting research
      - Creating creative content, such as banners or posters
      - Preparing presentations
      - Accessing information quickly
      - Preparing education or study plans
      - Other, please specify: __________
Section 3. Perceived Usefulness of AI Tool Functionalities
  4. Which features of AI tools do you find useful? Please rate each item on a scale from 1 to 5.
    (1 = Not useful at all to 5 = very useful)
      - Providing rapid feedback
      - Offering alternative suggestions
      - Guiding users toward accurate information
      - Providing writing and language support
      - Facilitating the content creation process
      - Providing support for visual materials
      - Saving time
      - Offering creative solutions
      - Other, please specify: __________
Section 4. NASA-TLX-Based Perceived Workload Ratings for the AI-Supported Pedagogical Design Task

Instructions. Please answer the following items by considering the lesson plan preparation task that you completed with the support of AI tools in your professional knowledge course. This task included designing an instructional activity and developing appropriate teaching materials. Please base your responses only on your experience while completing this specific task, not on your general academic workload or general AI use experiences. For each item, enter a score between 0 and 100.

1. Mental Demand: How much mental and perceptual activity was required while completing the AI-supported task of preparing a lesson plan, designing an instructional activity, and developing teaching materials? Consider processes such as thinking, decision-making, searching for information, comparing alternatives, remembering, and organizing information.
2. Physical Demand: How much physical activity or physical effort was required while completing this AI-supported lesson planning, instructional activity design, and material development task?
3. Temporal Demand: How much time pressure did you feel while completing this AI-supported task?
4. Performance: How successful do you think you were in achieving the goals of this AI-supported task? Consider the appropriateness of the lesson plan, the quality of the instructional activity, and the relevance of the developed teaching materials.
5. Effort: How much overall mental and physical effort was required to complete this AI-supported lesson planning, instructional activity design, and material development task?
6. Frustration: While completing this AI-supported task, how tense, stressed, confused, irritated, challenged, or discouraged did you feel?
